# Preclinical In Vitro Evaluation of Extracellular Vesicles from Human Dental Pulp Stem Cells for the Safe and Selective Modulation of Anaplastic Thyroid Carcinoma

**DOI:** 10.3390/ijms26136443

**Published:** 2025-07-04

**Authors:** Anderson Lucas Alievi, Michelli Ramires Teixeira, Vitor Rodrigues da Costa, Irina Kerkis, Rodrigo Pinheiro Araldi

**Affiliations:** 1 Postgraduate Program in Endocrinology and Metabology, Paulista School of Medicine of the Federal University of São Paulo (EPM/UNIFESP), São Paulo 04023-062, SP, Brazil; anderson.alievi@butantan.gov.br (A.L.A.); michelli.teixeira@butantan.gov.br (M.R.T.); 2 Genetics Laboratory, Butantan Institute, São Paulo 05503-900, SP, Brazil; vitor.rodrigues@butantan.gov.br; 3 Department of Morphology and Genetics, Paulista School of Medicine of the Federal University of São Paulo (EPM/UNIFESP), São Paulo 04023-062, SP, Brazil; 4 BioDecision Analytics Ltd., São Paulo 05713-510, SP, Brazil

**Keywords:** anaplastic thyroid carcinoma, extracellular vesicles, dental pulp stem cells, cell migration, tumor invasion, cancer nanomedicine

## Abstract

Anaplastic thyroid carcinoma (ATC) is a highly aggressive malignancy with poor prognosis and limited treatment options. Precision oncology seeks personalized therapies that selectively modulate tumor behavior, which is critical for improving patient outcomes. In this study, we evaluated the therapeutic potential of human dental pulp stem cell-derived extracellular vesicles (hDPSC-EVs) in three ATC cell lines (8505C, HTH83, KTC-2). Fluorescence and confocal microscopy confirmed the efficient, time-dependent internalization of hDPSC-EVs by ATC cells, with increased fluorescence intensity over 48 h. Functional assays revealed the selective inhibition of migration and invasion in a cell line-dependent manner, without affecting cell proliferation, viability, or tumorigenic traits, indicating a non-cytotoxic, context-specific modulation of tumor behavior. After 72 h of EV treatment, targeted qPCR of 92 cancer-related genes showed the strongest response in 8505C cells (24 genes; 16 up, 8 down), moderate changes in KTC-2 (16 genes; 14 up, 2 down), and few alterations in HTH83 (6 genes; 4 up, 2 down). Across all lines, *FN1* emerged as a context-dependent target, downregulated in 8505C but upregulated in the other two. No broad pathway enrichment was observed, indicating the fine-tuning of key networks rather than wholesale reprogramming. Despite variations across cell lines, hDPSC-EVs consistently demonstrated no impact on cell proliferation and no evidence of cytotoxicity or tumorigenic behavior, with no adverse outcomes. These findings provide preclinical evidence for hDPSC-EVs as a promising, safe, and targeted therapeutic platform in precision oncology, particularly for aggressive cancers, like ATC, warranting further exploration in preclinical and clinical studies.

## 1. Introduction

Anaplastic thyroid carcinoma (ATC) is one of the most aggressive and deadly malignancies, characterized by rapid progression, resistance to conventional therapies, and poor prognosis. While surgical resection, intensity-modulated radiation therapy (IMRT), and chemotherapy regimens offer marginal improvements in survival, these treatments remain ineffective in overcoming the profound clinical challenges posed by ATC [1,2,3,4,5]. Targeted therapies, such as tyrosine kinase inhibitors (e.g., sorafenib, lenvatinib), have shown limited efficacy, underscoring the urgent need for new therapeutic strategies to improve clinical outcomes [4,5].

Nanomedicine is a promising field, introducing nanotechnological platforms such as liposomes, micelles, and extracellular vesicles (EVs) for cancer treatment, enabling the delivery of various molecules, such as drugs, radioiodine, and genes [6,7]. Among these, mesenchymal stem cell-derived EVs (MSC-EVs) have emerged as especially promising: they shuttle a rich cargo of proteins, lipids, DNA, messenger RNA, and microRNAs; can modulate immune responses; and readily traverse biological barriers [8,9,10,11,12,13,14].

Human dental pulp stem cell (hDPSC)-derived EVs exhibit pro-angiogenic effects in endothelial cells and can influence immune cell behavior, properties that both support tissue repair and hold potential for anticancer activity [15,16,17]. Despite the encouraging results in regenerative and preclinical cancer models, the impact of hDPSC-EVs on extremely aggressive tumors, like ATC, has not yet been defined.

Importantly, our group has extensively characterized the hDPSCs used in this study over the past 25 years. These cells have more recently been utilized in three clinical studies (ClinicalTrials.gov identifiers NCT04315987, NCT02728115, and NCT03252535), all conducted under regulatory oversight, which have confirmed their favorable safety profile. The established clinical and regulatory experience with hDPSCs adds a layer of validation for the safety of their EV derivatives and further motivates their investigation in oncology. As one of the key mechanisms by which stem cells exert their therapeutic effects, EVs are central to mediating regenerative and immunomodulatory action. This long-standing preclinical and clinical validation provides a strong foundation for exploring the therapeutic potential of hDPSC-derived EVs in cancer treatment—particularly for aggressive malignancies, such as ATC, where treatment options remain limited and patient outcomes are poor.

Given their unique properties, extracellular vesicles derived from human dental pulp stem cells (hDPSC-EVs) may represent a promising strategy for modulating tumor behavior in anaplastic thyroid carcinoma (ATC). By targeting malignant features, such as migration and invasion, these vesicles can exert therapeutic effects without promoting cell proliferation or other oncogenic traits. In this study, we investigated the in vitro therapeutic potential of hDPSC-EVs in ATC, focusing on their ability to influence key tumor cell behaviors, including motility, invasiveness, and growth. To capture context-dependent responses, we used three common ATC cell lines (8505C, HTH83, and KTC-2) selected for their molecular diversity, including mutations in key genes, such as *BRAF^V600E^*, *TP53*, and *HRAS*. We aimed to reflect the major oncogenic profiles found in ATC with this panel, which supports the assessment of differential responses to hDPSC-EV treatment across distinct genetic backgrounds [18,19,20]. This research contributes to the growing body of evidence supporting MSC-EVs as a promising tool for cancer treatment, particularly in aggressive, treatment-resistant cancers, like ATC, where current therapeutic options are limited.

## 2. Results

### 2.1. Distribution, Particle Concentration, and Protein Content of hDPSC-EVs

The size distribution and particle concentration of hDPSC-EVs were analyzed using Nano Sight NS300, which revealed a mean particle size of 172.1 ± 46.3 nm [corresponding to ‘small EVs’ (<200 nm) according to the definition criteria proposed by ISEV] and a concentration of 1.13 × 10^9^ particles/mL (Figure S3). Protein quantification, performed using the BCA Protein Assay, showed a protein concentration of 649.31 ± 5.80 μg/mL.

### 2.2. Uptake of hDPSC-EVs by ATC Cell Lines

**Figure 1:**
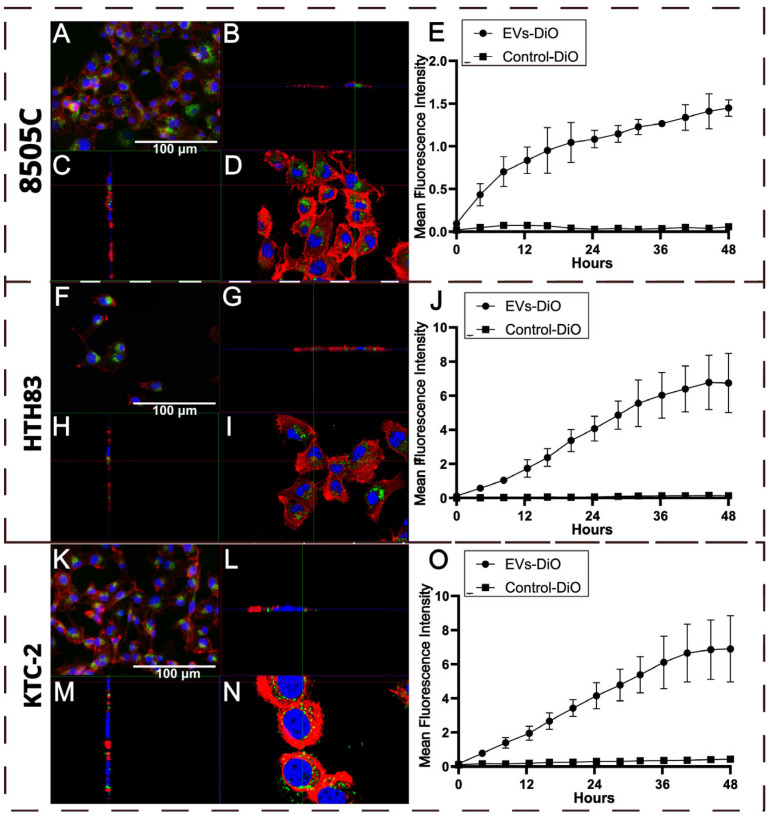
Uptake of fluorescently labeled EVs (EVs-DiO) by ATC cell lines 8505C, HTH83, and KTC-2. Representative 2D conventional fluorescence microscopy images (**A**,**F**,**K**) show the apparent uptake of EVs-DiO (green) after 24 h of incubation. Orthogonal views obtained via confocal fluorescence microscopy (**B**–**D**,**G**–**I**,**L**–**N**) confirm the internalization of EVs-DiO by these cell lines after 24 h. Graphs (**E**,**J**,**O**) display the MFI of EVs-DiO in each cell line over 48 h, measured by time-lapse confocal microscopy. Control cells incubated with Control-DiO were used to establish baseline fluorescence levels in the time-lapse confocal experiment. In representative 2D and 3D images, cell nuclei are counterstained with DAPI (blue), and F-actin is stained with Alexa Fluor™ 647 Phalloidin (red). Scale bar = 100 μm. In graphs (**E**,**J**,**O**), the data are presented as the mean ± standard deviation (SD) from three independent wells of independent experiments.

The uptake of fluorescently labeled hDPSC-EVs (EVs-DiO) by ATC cell lines 8505C, HTH83, and KTC-2 was assessed using fluorescence and confocal microscopy. Representative 2D fluorescence microscopy images (Figure 1A,F,K) demonstrated the successful internalization of EVs-DiO (green) after 24 h of incubation. Confocal microscopy provided orthogonal views (Figure 1B–D,G–I,L–N), which confirmed the presence of EVs-DiO within the cytoplasm, offering a more detailed, three-dimensional perspective of their internalization.

Quantitative analysis of the mean fluorescence intensity (MFI) over 48 h (Figure 1E,J,O) revealed a time-dependent increase in fluorescence, consistent with sustained EV uptake. Control cells treated with dye alone (Control-DiO) exhibited negligible fluorescence, confirming that the signal originated specifically from internalized EVs rather than dye aggregates.

### 2.3. Transcriptomic Results of RNA Sequencing and qPCR Array

To investigate the molecular effects of hDPSC-EVs on anaplastic thyroid carcinoma cells, we performed RNA sequencing (RNA-seq) 72 h after treatment. All RNA-seq reads underwent quality assessment using FastQC and MultiQC software, with summary metrics provided in Table S2. Although our study initially included all three ATC cell lines, only 8505C and KTC-2 were retained for transcriptomic analysis. The HTH83 samples were excluded due to insufficient RNA integrity; specifically, they did not meet the quality threshold (RIN ≥ 7) required for library preparation, and their inclusion could have introduced technical bias. Differential gene expressions were analyzed through gene set enrichment analysis (GSEA), using the Reactome database to identify significantly modulated pathways.

**Figure 2:**
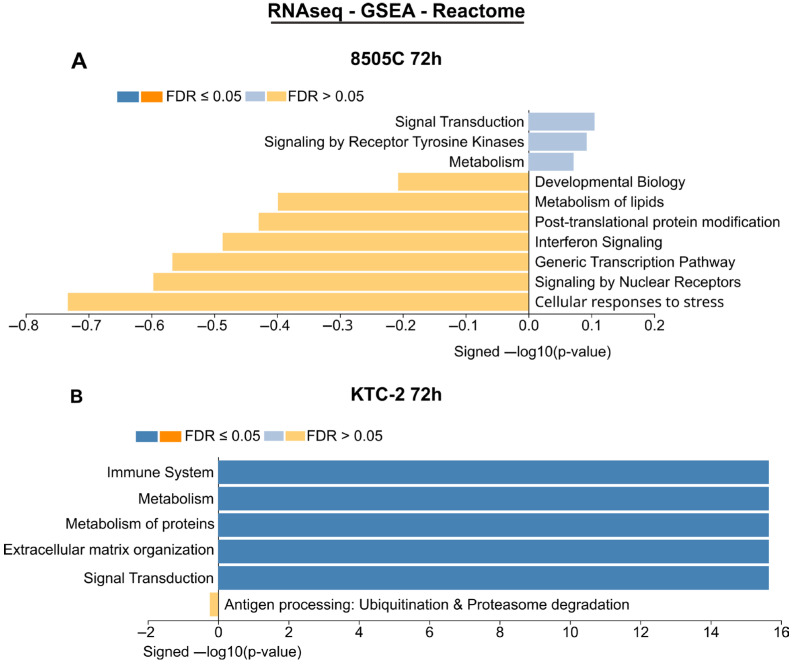
RNA-seq results. The figure shows the gene set enrichment analysis (GSEA) of differentially expressed genes (DEGs) in the anaplastic thyroid cancer (ATC) cell lines 8505C and KTC-2 after 72 h of treatment with hDPSC-EVs. GSEA was performed using the Reactome database as a reference. Bar graphs represent the top biological pathways enriched in each cell line, sorted by signed-log10 (*p*-value). Only pathways with a false discovery rate (FDR) ≤ 0.05 are considered significantly enriched (deep blue bars). Pathways with FDR > 0.05 (yellow bars and faint blue bars) are shown for reference of the top non-enriched terms. (**A**) The 8505c cells did not show significant enrichment in any pathway. (**B**) KTC-2 cells showed enrichment in pathways related to the immune system, metabolism, organization of the extracellular matrix, and signal transduction.

In the 8505C cell line, gene set enrichment analysis (GSEA) at 72 h post-treatment with hDPSC EVs revealed no pathways meeting the significance threshold (FDR ≤ 0.05) (Figure 2A). By contrast, KTC 2 cells displayed a robust, positive enrichment of multiple cancer-related pathways (FDR < 0.05), notably those governing immune responses, protein metabolism, and extracellular matrix organization (Figure 2B). No pathways were significantly downregulated in KTC 2. Together, these data indicate that hDPSC EVs selectively modulate key tumor-associated and immunomodulatory processes in KTC 2 cells while exerting a minimal transcriptional impact on 8505C cells.

To further validate and extend the RNA-seq findings, we performed quantitative PCR (qPCR) using the TaqMan™ Array Human Molecular Mechanisms of Cancer (Applied Biosystems™, Waltham, MA, USA), targeting 92 genes implicated in key cancer-related pathways. This analysis was conducted on three ATC cell lines (8505C, HTH83, and KTC-2) after 72 h of treatment with hDPSC-EVs. Gene expression changes with log_2_FC ≥ 0.58 (upregulated) or ≤−0.58 (downregulated) were considered biologically relevant and are presented as individual bar graphs for each cell line.

**Figure 3:**
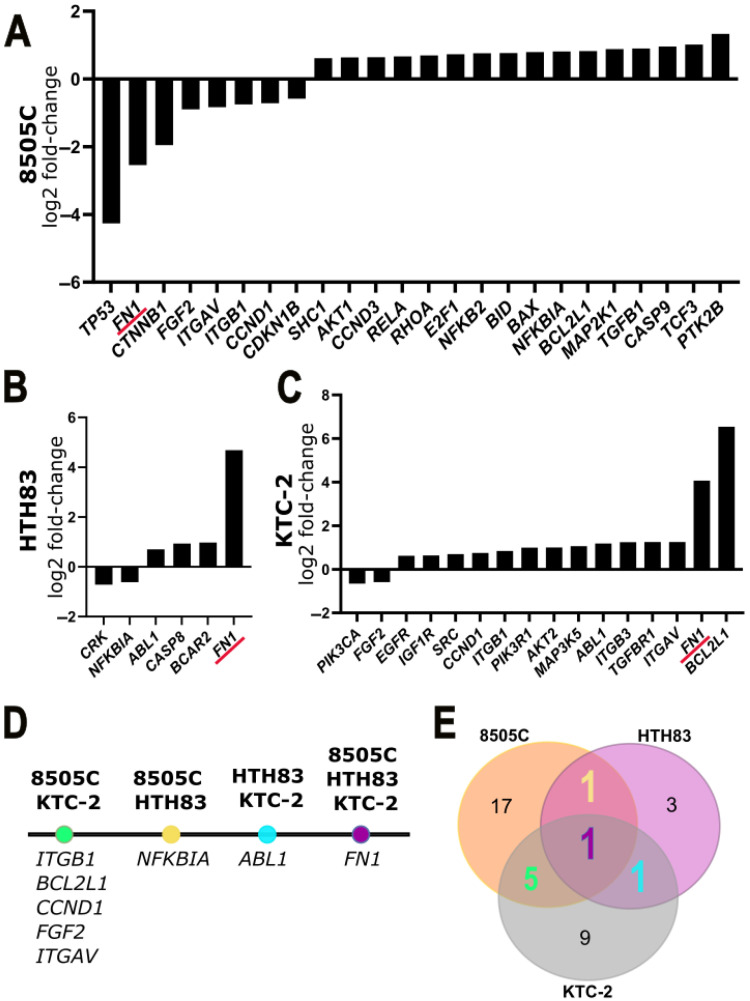
Differential mRNA expression of selected genes in three ATC cell lines. (**A**) Bar graph of log_2_ fold-changes in 8505C cells, ordered from strongest downregulation (*TP53*) to strongest upregulation (*PTK2B*). (**B**) Bar graph of six transcripts in HTH83 cells, showing the downregulation of *CRK* and *NFKBIA* and induction of *FN1*. (**C**) Log_2_ fold-changes for 14 genes in KTC-2 cells, with pronounced increases in *BCL2L1* and *FN1*. (**D**) Schematic of pairwise overlaps: five genes shared by 8505C and KTC-2 (*ITGB1*, *BCL2L1*, *CCND1*, *FGF2*, *ITGAV*), one between 8505C and HTH83 (*NFKBIA*), one between HTH83 and KTC-2 (*ABL1*), and *FN1* common to all three lines. (**E**) Venn diagram quantifying unique and shared differentially expressed genes: 17 exclusives to 8505C, 3 to HTH83, 9 to KTC-2, and the overlaps detailed in (**D**) Red underline emphasizes *FN1*’s consistent modulation across all three cell lines.

8505C cells exhibited the greatest transcriptional response, with 24 differentially expressed genes (DEGs; 16 upregulated, 8 downregulated), followed by KTC-2 cells (16 DEGs; 14 upregulated, 2 downregulated), and HTH83 cells (6 DEGs; 4 upregulated, 2 downregulated) (Figure 3B–D).

In 8505C cells, hDPSC-EVs selectively modulated key signaling pathway genes. *TP53*, *FN1*, *CTNNB1*, *FGF2*, *ITGAV*, *ITGB1*, *CCND1*, and *CDKN1B* were significantly downregulated, while genes involved in cell cycle and survival (*AKT1*, *CCND3*, *E2F1*, *RELA*), apoptosis (*BID*, *BAX*, *CASP9*), *MAPK* and growth signaling (*MAP2K1*, *SHC1*), transcriptional regulation (*TGFB1*, *TCF3*), and inflammation and cytoskeletal dynamics (*NFKB2*, *RHOA*, *PTK2B*, *NFKBIA*, *BCL2L1*) were upregulated. The pronounced downregulation of *TP53* in 8505C cells, which harbor a non-functional *TP53* mutation, is unlikely to impact tumor suppressor activity or increase oncogenic risk, maintaining the safety profile of the EVs

In HTH83 cells, hDPSC-EVs modulated fewer genes, downregulating *CRK* and *NFKBIA*, involved in cytoskeletal and inflammatory pathways, respectively, and upregulating *FN1*, *BCAR2*, *CASP8*, and *ABL1*, associated with extracellular matrix remodeling, cell survival, apoptosis, and signal regulation.

In KTC-2 cells, hDPSC-EVs altered gene expression, upregulating genes related to apoptosis regulation (*BCL2L1*), integrin signaling (*ITGAV*, *ITGB3*, *ITGB1*), growth and survival signaling (*ABL1*, *AKT2*, *SRC*, *EGFR*, *CCND1*, *IGF1R*), *PI3K* signaling (*PIK3R1*), TGF-beta signaling (*TGFBR1*), and *MAPK* signaling (*MAP3K5*), while downregulating genes involved in growth factor and angiogenic signaling (*PIK3CA*, *FGF2*).

A Venn diagram illustrates the genes consistently modulated across the three ATC cell lines, highlighting *FN1* as a core target with distinct expression patterns, upregulated in HTH83 and KTC-2, but downregulated in 8505C, emphasizing the highly context-dependent nature of hDPSC-EV-mediated gene regulation. Gene set enrichment analysis of the qPCR data did not identify significantly enriched pathways (FDR ≤ 0.05), suggesting the targeted fine-tuning of specific gene networks rather than the broad reprogramming of canonical cancer pathways. The full list of enriched pathways and their corresponding FDR values is provided in Figure S4.

Together, these results highlight the complexity and specificity of the cellular response to hDPSC-EV treatment. The identification of common and cell line-specific gene expression changes suggests a context-dependent mechanism of action. Further exploration of the consistently modulated genes, particularly *FN1*, *PIK3R1*, and *TGFBR1*, may reveal new molecular insights and therapeutic targets for managing aggressive thyroid cancers. A full list of DEGs and associated enrichment analyses is provided in Table S3.

### 2.4. hDPSC-EVs Do Not Alter Viability, Cell Cycle Progression, or Proliferation in ATC Cell Lines

**Figure 4:**
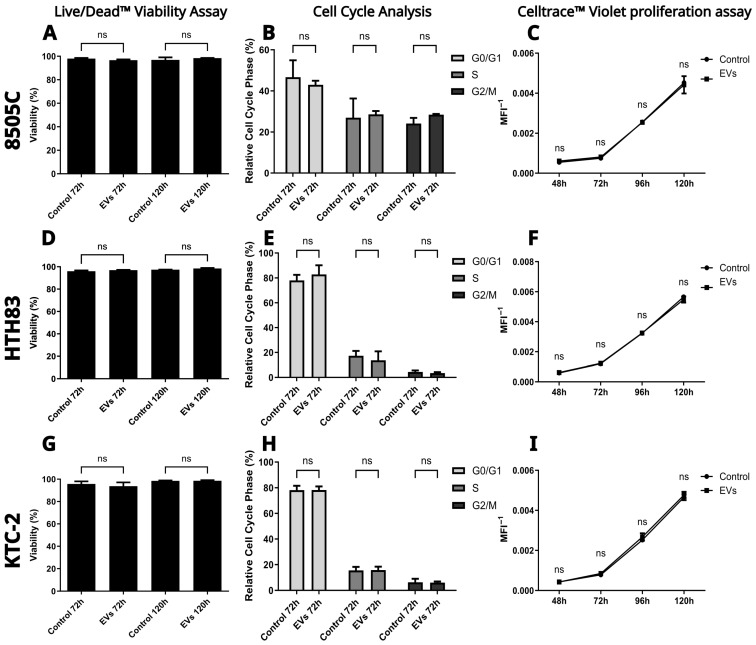
Effect of hDPSC-EVs on the viability, cell cycle progression, and proliferation of ATC cell lines. In the Live/Dead™ viability assay (**A**,**D**,**G**), the viability of 8505C, HTH83, and KTC-2 cell lines was assessed after 72 h and 120 h of incubation with and without hDPSC-EVs. No significant differences were observed between the control and EV-treated groups at either time point (Mann–Whitney test, *p* > 0.05). In the cell cycle analysis (**B**,**E**,**H**), the cell cycle distribution of ATC cell lines was analyzed by flow cytometry after 72 h of incubation. No significant differences were observed between the control and EV-treated groups in the percentage of cells in G0/G1, S, or G2/M phases (Mann–Whitney test for each cell cycle phase between treatments, *p* > 0.05). In the CellTrace™ Violet proliferation assay (**C**,**F**,**I**), the proliferation of ATC cell lines was monitored over time (48 h, 72 h, 96 h, 120 h) using CellTrace™ Violet dye. The mean fluorescence intensity (MFI) of the dye, which decreases as cells divide, was measured by flow cytometry. No significant differences in proliferation were observed between the control and hDPSC-EV-treated groups at any time point (Mann–Whitney test, *p* > 0.05). ns: non-significant.

To assess the impact of hDPSC-EVs on cell viability, we conducted a Live/Dead™ viability assay (Figure 4A,D,G) across ATC cell lines following 72 and 120 h of incubation with or without hDPSC-EVs. The analysis revealed no significant differences in viability between the control and EV-treated groups at either time point (Mann–Whitney test, *p* > 0.05). These results indicate that hDPSC-EVs do not exert cytotoxic effects under the tested conditions.

Cell cycle progression was evaluated using the FxCycle™ PI/RNase Staining Solution, a propidium iodide-based assay, followed by flow cytometry after 72 h of treatment (Figure 4B,E,H). The distribution of cells across G0/G1, S, and G2/M phases showed no significant differences between the control and EV-treated groups (Mann–Whitney test, *p* > 0.05 for all phases), suggesting that hDPSC-EVs do not alter cell cycle dynamics in ATC cell lines.

To investigate potential effects on cell proliferation, we used the CellTrace™ Violet assay (Figure 4C,F,I), which monitors proliferation by measuring MFI via flow cytometry. As cells divide, dye dilution results in reduced fluorescence. Proliferation was tracked over 48, 72, 96, and 120 h, and no significant differences were observed between the control and EV-treated groups at any time point (Mann–Whitney test, *p* > 0.05). These findings suggest that hDPSC-EVs do not significantly affect the proliferative capacity of ATC cell lines.

Due to high confluence at the 120 h mark, further analysis beyond this point was not pursued. At extended incubation times, confluence-related changes and medium acidification may compromise cellular physiology, introducing variability that could obscure the effects of hDPSC-EVs.

### 2.5. Effects on Mitochondrial Function and ROS Production

**Figure 5:**
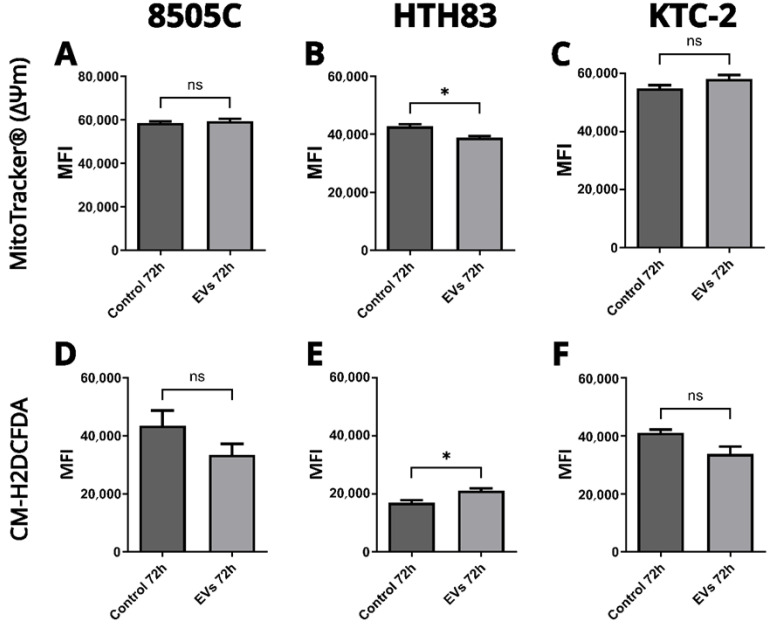
Assessment of mitochondrial function and ROS production in cells treated with EVs. The mitochondrial membrane potential (ΔΨm) was measured using MitoTracker^®^ (**A**–**C**), and ROS levels were measured using CM-H2DCFDA (**D**–**F**) after 72 h of incubation with or without hDPSC-EVs. Statistical significance was determined using a Mann–Whitney test (* *p* < 0.05; ns: non-significant).

Mitochondrial membrane potential (ΔΨm) was evaluated using MitoTracker^®^ staining (Figure 5A–C) to assess the impact of hDPSC-EVs on mitochondrial function. In the HTH83 cell line, treatment with hDPSC-EVs induced a small but statistically significant reduction in ΔΨm compared to the control group (Mann–Whitney test, *p* < 0.05), suggesting a potential influence on mitochondrial activity. In contrast, no significant changes in ΔΨm were observed in the 8505C and KTC-2 cell lines following hDPSC-EV treatment (Mann–Whitney test, *p* > 0.05). These findings indicate that the effects of hDPSC-EVs on mitochondrial function are cell line-specific, with HTH83 cells showing a distinct response.

Reactive oxygen species (ROS) levels were measured using the CM-H2DCFDA probe (Figure 5D–F) to evaluate oxidative stress. hDPSC-EV treatment resulted in a slight but significant increase in ROS production in HTH83 cells compared to the controls (Mann–Whitney test, *p* < 0.05), indicating enhanced oxidative stress. However, no significant differences in ROS levels were detected in the 8505C and KTC-2 cell lines (Mann–Whitney test, *p* > 0.05). These results further support the notion that hDPSC-EV-mediated effects on mitochondrial function and oxidative stress are specific to the HTH83 cell line, with no observable impact on 8505C and KTC-2 cells under the same conditions.

### 2.6. Inhibition of Migration and Invasion by hDPSC-EVs in ATC Cells

The migratory capacity of ATC cell lines 8505C, HTH83, and KTC-2 was assessed using a scratch wound assay to evaluate the effect of hDPSC-EVs on cell migration. Wound closure was monitored from 0 to 48 h for 8505C cells and from 0 to 36 h for HTH83 and KTC-2 cells. Treatment with hDPSC-EVs significantly impaired wound closure in KTC-2 cells at 24, 28, and 32 h, and in HTH83 cells between 8 and 20 h, compared to their respective controls. In contrast, no significant differences were observed in the 8505C cell line under the same experimental conditions. Statistical analysis was performed using multiple unpaired *t*-tests followed by Šídák correction (*p* < 0.05). These findings suggest that hDPSC-EVs selectively inhibit the migratory capacity of KTC-2 and HTH83 cells, indicating a potential therapeutic effect on cell motility in these ATC subtypes.

**Figure 6:**
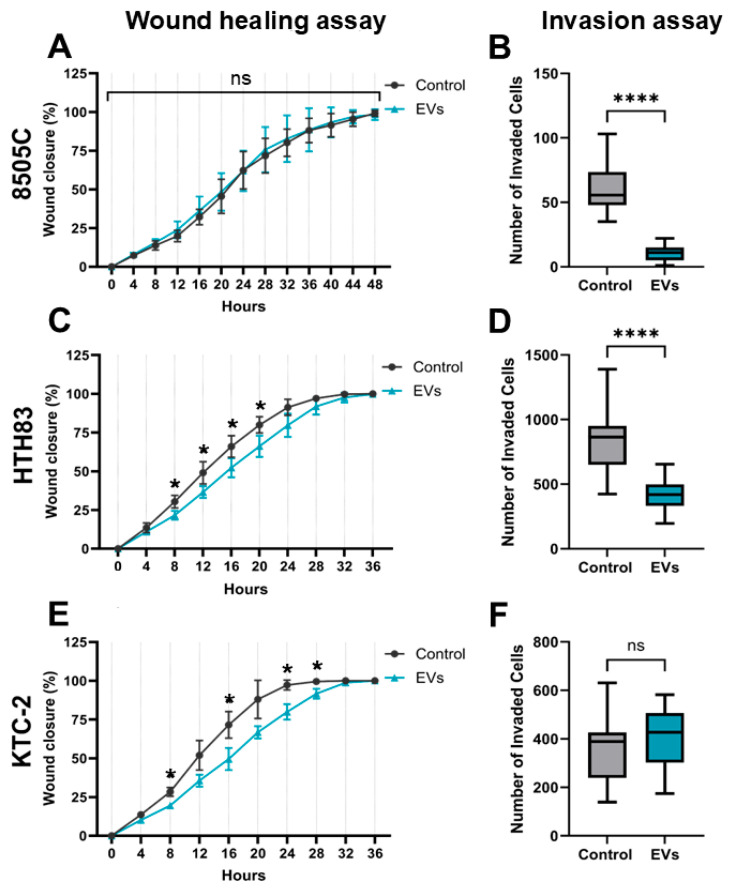
Effect of hDPSC-EVs on the wound healing and invasion capacity of ATC cell lines. Wound healing assay (**A**,**C**,**E**): The migratory capacity of 8505C, HTH83, and KTC-2 cells was assessed using a scratch wound assay. The percentage of wound closure was monitored over time (0 h–48 h for 8505C, 0h–36h for HTH83 and KTC-2). hDPSC-EV treatment significantly decreased wound closure in KTC-2 cells at 24 h, 28 h, and 32 h, and in HTH83 cells between the 8 h and 20 h time points, while no significant effect was observed on 8505C cells (multiple unpaired *t*-tests followed by the Šídák multiple comparisons test; * *p* < 0.05). Invasion assay (**B**,**D**,**F**): The invasive capacity of ATC cells was evaluated using a Transwell invasion assay with a Matrigel matrix. Cells were treated with 50 µg/mL of hDPSC-EV protein mass-relative for 72 h before the assay. The number of invaded cells was quantified after 24 h. hDPSC-EV treatment significantly decreased the invasive capacity of 8505C and HTH83 cells, while no significant effect was observed on KTC-2 cells (unpaired *t* test; **** *p* < 0.0001). Representative images of the wound healing and invasion assays are provided in Figures S5 and S6, respectively. ns: non-significant.

The invasive potential of ATC cell lines was further evaluated using a Transwell invasion assay with a Matrigel matrix (Figure 6). Cells were pretreated with 50 µg/mL of hDPSC-EVs (based on protein content) for 72 h prior to the assay, and the number of invaded cells was quantified after 24 h. hDPSC-EV treatment significantly reduced the invasive capacity of 8505C and HTH83 cells compared to the controls (*t*-test, *p* < 0.0001), whereas no significant effect was observed on KTC-2 cells.

Collectively, these results highlight the differential effects of hDPSC-EVs on ATC cell behavior. While hDPSC-EVs inhibit both migration and invasion in HTH83 cells, and selectively impair invasion in 8505C cells, they appear to affect only migration (but not invasion) in KTC-2 cells. These findings underscore the context-dependent nature of hDPSC-EV activity and suggest a cell line-specific therapeutic potential targeting motility-associated processes in ATC.

## 3. Discussion

Fluorescence and confocal microscopy confirmed the efficient and time-dependent uptake of hDPSC-EVs by all three ATC cell lines (8505C, HTH83, and KTC-2), demonstrating successful delivery and cellular engagement. Functional assays revealed that hDPSC-EVs selectively impaired migration and invasion in a cell line-dependent manner, without promoting proliferation, viability, or tumorigenic traits. This suggests a context-specific, non-cytotoxic modulation of tumor behavior, supporting the safety and therapeutic potential of hDPSC-EVs.

Transcriptomic profiling via qPCR revealed that 8505C cells displayed the most extensive response to hDPSC-EVs (24 DEGs; 16 up, 8 down), followed by KTC-2 (16 DEGs; 14 up, 2 down), and HTH83 (6 DEGs; 4 up, 2 down). Despite the regulation of key genes, no pathway reached significant enrichment (FDR ≤ 0.05), underscoring a mechanism of fine-tuning specific gene networks rather than global reprogramming. The lack of statistically enriched pathways, especially in 8505C cells, may be explained by the relatively modest fold changes observed and the limited coverage of the 92-gene qPCR panel, both of which reduce the sensitivity and scope of enrichment analyses. Even so, some sub-threshold signals detected in both the RNA-seq and qPCR datasets suggest the possible modulation of biological processes, such as extracellular matrix organization, integrin-mediated adhesion, and apoptotic regulation, which may merit further investigation in future studies. Although our transcriptomic analyses revealed the cell line-specific modulation of genes associated with motility and signaling, the present data do not permit conclusive mechanistic inferences. Additional studies are needed to validate causal relationships and downstream pathway activation.

The interaction of hDPSC-EVs with the three ATC cell lines revealed context-dependent effects on key pathways, including apoptosis, *MAPK*/*ERK* signaling, and cell–matrix adhesion. In 8505C cells, the simultaneous downregulation of extracellular matrix and integrin genes (*FN1*, *ITGAV*, *ITGB1*) and upregulation of survival/apoptotic regulators (*AKT1*, *CCND3*, *E2F1*) aligns with reduced invasion but intact 2D migration [21,22,23,24,25,26]. *FN1*, which encodes fibronectin, plays a central role in cell adhesion and migration; its downregulation may contribute to impaired extracellular matrix interactions and reduced invasive capacity [22,27]. Although *TP53* was among the most downregulated genes in 8505C cells treated with hDPSC-EVs, this cell line harbors a non-functional *TP53* mutation. Therefore, the reduction in transcript levels is unlikely to further impair tumor suppressor function. The lack of proliferative or tumorigenic effects in functional assays supports this interpretation and underscores the safety profile of the EV treatment.

In contrast, KTC-2, the upregulation of integrin subunits (*ITGAV*, *ITGB3*, *ITGB1*), and *FN1* may preserve the 3D invasive capacity [28]. HTH83 cells exhibited a coordinated downregulation of *CRK* and *NFKBIA*, correlating with decreases in both invasion and migration assays [29,30].

Despite these variations, our results show that hDPSC-EVs did not induce cytotoxicity, alter cell cycle progression, or promote cell proliferation in any of the three ATC cell lines tested, indicating a consistent lack of proliferative or tumorigenic effects, with no adverse outcomes observed in functional assays. These findings align with the previous in vitro results from our group [31] and support the potential of hDPSC-EVs as a safe, biologically active alternative to cytotoxic drugs. These advantages are further underscored by the clinical track record of parental hDPSCs, which have been evaluated in multiple regulated human trials (ClinicalTrials.gov identifiers NCT04315987, NCT02728115, and NCT03252535). These studies have consistently demonstrated safety, tolerability, low immunogenicity, an absence of tumorigenicity, and no ectopic tissue formation. This robust translational background, combined with the acellular and non-replicative nature of hDPSC-EVs, suggests that these vesicles inherit the favorable safety profile of their cells of origin and offer significant advantages over live cell therapies. These advantages are consistent with the findings from other studies using MSC-EVs in cancer models, where similar properties, such as a reduction in cell migration, invasion, efficient tumor tropism, and the ability to modulate cell signaling, have been reported [32,33,34]. While the mechanisms of action may vary according to tumor context and EV source, the use of EVs from clinically validated stem cell platforms, such as hDPSCs, represents a promising extension of this strategy.

While our findings are promising, there are a few limitations to consider. First, we tested hDPSC-EVs in a limited number of ATC cell lines, which may not fully represent the diversity of ATC tumors. Future studies should include a broader range of cell lines to confirm the generalizability of our results. Second, our study was conducted in vitro, and the results may differ in vivo due to the complexity of the tumor microenvironment. Preclinical animal models will be essential to assess the biodistribution, safety, and efficacy of hDPSC-EVs in a more physiologically relevant context. Finally, although previous clinical trials have established the safety of hDPSCs, ensuring consistent EV preparation and quality control at scale will be crucial for their clinical application.

In conclusion, hDPSC-EVs hold substantial promises for targeted therapy in ATC within the context of precision oncology, offering the ability to modulate tumor behavior safely and selectively. Their cell line-dependent activity, lack of toxicity, established clinical safety, and favorable regulatory background position them as a compelling, scalable platform for personalized cancer treatment. Moreover, due to their natural biocompatibility and efficient cellular uptake, hDPSC-EVs also represent a versatile vehicle for the delivery of anticancer drugs, potentially enhancing therapeutic specificity while minimizing systemic toxicity. Future studies will focus on dissecting the molecular mechanisms underlying the observed phenotypes using pathway inhibition, gene editing, and proteomic approaches. Nonetheless, further investigation into their molecular mechanisms and in vivo preclinical validation is essential to support their safe and effective translation into clinical applications.

## 4. Materials and Methods

### 4.1. Cell Culture Conditions and Treatments

The anaplastic thyroid carcinoma cell lines 8505C, HTH83, and KTC-2 were cultured in 75 cm^2^ flasks (Corning^®^, Corning, NY, USA). The culture medium used was RPMI 1640 (Gibco™, Grand Island, NY, USA) supplemented with 10% fetal bovine serum (FBS) for the 8505C and HTH83 cell lines and 5% FBS for the KTC-2 cell line (Gibco™, Grand Island, NY, USA). An antibiotic solution of penicillin/streptomycin (Gibco™, Grand Island, NY, USA) was added to the medium to obtain final concentrations of 100 U/mL and 100 μg/mL, respectively. The medium was refreshed every three to four days. Upon reaching 70–80% confluence, cells were washed with PBS (Gibco™, Grand Island, NY, USA) and detached using the TrypLE™ Select enzyme (Gibco™, Grand Island, NY, USA). The cells were plated according to the specific requirements of each experiment. In each assay, the cell lines were exposed to 50 μg/mL of hDPSC-EV relative protein mass, in addition to a negative control consisting of only the complete culture medium. Additional controls were established as needed, and details are provided in specific experimental sections.

### 4.2. Human Dental Pulp Stem Cells’ Culture and Characterization

Human dental pulp stem cells (hDPSCs) were isolated as described by Kerkis et al. [35]. Isolated hDPSCs were cultured in DMEM/F-12 medium (Gibco, Grand Island, NY, USA) supplemented with 10% fetal bovine serum (FBS) (Corning, Corning, NY, USA), 100 U/mL penicillin, and 100 μg/mL streptomycin (Gibco, Grand Island, NY, USA). Cells were maintained under standard culture conditions at 37 °C in a humidified atmosphere containing 5% CO_2_ and passaged upon reaching 80–90% confluence until the fifth passage. Flow cytometry was used to characterize the hDPSCs based on their surface marker expression. The cells were analyzed for the presence of mesenchymal stem cells markers and the absence of hematopoietic markers. Cells were harvested at 80–90% confluence using the TrypLE™ Select enzyme (Gibco™, Grand Island, NY, USA), washed with PBS, and fixed using a 4% paraformaldehyde solution in PBS (PFA 4%). Approximately 5 × 10^5^ cells were resuspended in 100 µL of PBS containing 1% BSA. The cells were then incubated with the following fluorochrome-conjugated antibodies for 30 min at 4 °C in the dark: CD105–APC, CD73–BB515, and CD90–APC as positive markers; CD45–BB515, CD34–APC, CD14–PerCP, CD19–APC, and HLA–DR-BB515 as negative markers (complete antibody information is provided in Table S1). After incubation, the cells were washed twice with PBS containing 1% BSA and resuspended in 300 µL PBS. Data acquisition was performed using a BD FACSCanto II flow cytometer (BD Biosciences, San Jose, CA, USA) and data analysis was conducted using FlowJo™ 10 software (BD, Ashland, OR, USA) using the gate strategy demonstrated in Figure S1A. The results from the characterization of hDPSCs are available in Figure S2.

### 4.3. Conditioned Medium Collection and hDPSC-EV Isolation

hDPSCs were cultured up to the fifth passage in HYPERFlask^®^ M Cell Culture Vessels (Corning^®^, Corning, NY, USA). Upon reaching 80% confluence, the culture medium was removed, and the cells were washed twice with PBS (Gibco™, Grand Island, NY, USA). Subsequently, the hDPSCs were incubated for 24 h in 560 mL of serum-free DMEM/F12 medium. Conditioned culture medium (CCM) was collected in sterile bottles. CCM clarification was performed using Stericup^®^ filters with a pore size of 0.22 μm (Millipore^®^, Burlington, MA, USA). Subsequently, the CCM was transferred to 13 mL polyamide ultracentrifuge tubes (Eppendorf Himac, Hitachinaka, Ibaraki, Japan) and ultracentrifuged at 100,000× *g* for 1 h in a Himac CP80WX ultracentrifuge (Eppendorf Himac, Hitachinaka, Ibaraki, Japan) using the P40ST rotor (Eppendorf Himac, Hitachinaka, Ibaraki, Japan), as described by Rajendran et al. (2021) [36]. The supernatant was carefully removed, and the hDPSC-EVs pellet was washed with PBS and ultracentrifuged again under the same conditions. The hDPSC-EVs pellet was resuspended in 0.9% NaCl solution (saline), filtered through a 0.22 μm syringe filter (Millipore^®^, Burlington, MA, USA), and stored in small aliquots at −80 °C for future use.

### 4.4. Determination of Size Distribution, Concentration, and Protein Mass-Relative of hDPSC-EVs

The size distribution and concentration of hDPSC-EVs were evaluated using NanoSight NS300 (Malvern Panalytical, Malvern, Worcestershire, UK) and NanoSight NTA v3.3 software (Malvern Panalytical, Malvern, Worcestershire, UK), an instrument used for nanoparticle tracking analysis (NTA). Initially, 50 μL of EVs was diluted in 950 μL of deionized water. Three captures of 60 s each were performed for subsequent analysis. Another quantification method used was measuring the relative hDPSC-EV protein mass, conducted using the Bicinchoninic Acid (BCA) Protein Assay. Total proteins were extracted using a RIPA buffer (Thermo Scientific™, Waltham, MA, USA) and analyzed in triplicate using a BCA kit (Thermo Scientific™, Waltham, MA, USA), according to the manufacturer’s recommendations. The analysis was performed using an Infinite^®^ 200 PRO microplate reader (Tecan, Männedorf, Switzerland) at a wavelength of 562 nm. Due to limited EV yield and a lack of access to equipment for Western blot or flow cytometry, we were unable to perform additional marker-based characterization. However, the applied methods are consistent with MISEV2023 recommendations in contexts where sample quantity is limited. This limitation has been acknowledged, and future studies will include EV marker profiling to complement these results.

### 4.5. Fluorescent Labeling of EVs

For the labeling of EVs, 200 μL of isolated hDPSC-EVs (~130 mg of protein mass-relative) was incubated with 1 μL of Vybrant™ DiO dye (Invitrogen™, Waltham, MA, USA) for 20 min at 37 °C. After incubation, 1 mL of 1% BSA solution in PBS was added and gently mixed by pipetting for one minute. PBS was added to complete the volume required for the ultracentrifuge tube (approximately 11.5 mL). Ultracentrifugation was performed as previously described. After the first centrifugation, the supernatant was discarded, and the pellet was resuspended in a sufficient volume of PBS to fill the tube (approximately 13 mL) and ultracentrifuged again under the previously described conditions. The supernatant was discarded, and the pellet was resuspended in 200 μL of 0.9% saline solution, filtered through a 0.22 μm filter (Millipore^®^, Burlington, MA, USA), and stored in small aliquots at −80 °C for future applications. This suspension of fluorescently labeled hDPSC-EVs was termed EVs-DiO. To ensure the specificity of labeling, a negative control was prepared simultaneously by subjecting only the diluent to the same process without the presence of EVs. This control was termed Control-DiO and used in uptake assays to confirm that the observed signals were exclusively attributable to the labeled hDPSC-EVs and not to artifacts such as dye micelles.

### 4.6. Uptake Assay by Fluorescence Microscopy and Confocal Microscopy

Microscopic analysis was performed to acquire 2D and 3D images of fluorescent EVs within the cytoplasm of cancer cell lines. Initially, 10,000 cells of each cell line were cultured per well in an 8-well Nunc LabTek chamber slide (Thermo Scientific, Waltham, MA, USA). After cell adhesion, the cell lines were treated with 50 μg/mL EVs-DiO or the corresponding volume of Control-DiO for 24 h. The cells were washed with PBS and fixed with PFA 4% for 20 min. Next, the cells were washed and stained with phalloidin–Alexa Fluor 647 (Thermo Scientific, Waltham, MA, USA) at a dilution of 1:400 in PBS for 1 h at room temperature (20–25 °C). Cells were washed twice with PBS, and the nuclei were stained with 1 µg/mL of DAPI (SigmaAldrich, Saint Louis, MO, USA) in PBS for 5 min. After the final washes with PBS, the chamber structure was removed from the slide and mounted using the ProLong™ Glass Antifade Mountant (Thermo Scientific™, Waltham, MA, USA) and a coverslip. The samples were initially examined under a conventional fluorescence microscope (Nikon Eclipse Ti, Tokyo, Japan) and subsequently under a Leica TCS SP8 Laser Confocal Microscope (Leica Microsystems, Wetzlar, Germany) equipped with a 63×/1.20 water immersion apochromatic objective, using LAS X office software (version 1.4.7, Leica Microsystems, Wetzlar, Germany) for 2D and 3D image acquisitions of each cell line.

### 4.7. Time-Lapse Confocal Microscopy Uptake Assay

To evaluate the internalization of labeled EVs over time, ATC cell lines were cultured in Black Nunc MicroWell 96-Well Optical-Bottom plates (Thermo Scientific, Waltham, MA, USA) and seeded at a density of 3 × 10^3^ cells per well in triplicate. After cell adhesion, the medium was replaced with a culture medium containing 50 µg/mL of EVs-DiO or Control-DiO. Captures were performed using a TCS SP8 confocal microscope (Leica Microsystems) with LAS X software (Leica Microsystems) every 45 min over 48 h (totaling 64 captures per well), recording multiple sections along the *Z*-axis to cover the full cell depth. The obtained images were analyzed using a macro from FIJI software (version 2.15.1, National Institutes of Health, Bethesda, MD, USA) to calculate the MFI of each field of EVs-DiO and Control-DiO over time (available at https://github.com/MichelliRT/EVsUptakeTimeLapseConfocal/tree/main, accessed on 23 May 2024). For MFI quantification, one field of view per well (of independent experiments) was analyzed across three independent wells per condition. Graphical representations of the data were created using GraphPad Prism version 10.0.0 (GraphPad Software, Boston, MA, USA).

### 4.8. RNA Extraction and Quality Control

For RNA extraction, cells were cultured in 25 cm^2^ flasks (Corning^®^, Corning, NY, USA) and treated with EVs, as previously described. After 72 or 120 h of treatment, the medium was removed and the cells were washed twice with PBS. Then, 1 mL of TRIzol™ Reagent (Invitrogen™, Waltham, MA, USA) was added to the cells. The cell lysate was transferred to DNase/RNase-free microtubes and mixed with 0.2 mL of chloroform by inversion for 10 min, followed by centrifugation at 10,000× *g* for 15 min at 4 °C. The aqueous phase was transferred to a new microtube and mixed via inversion with isopropanol (0.5 mL) for 10 min at 4 °C. After centrifugation at 10,000× *g* for 30 min at 4 °C, the supernatant was discarded. The RNA pellet was washed three times with 1 mL of cold 90% ethanol, with each wash consisting of centrifugation at 10,000× *g* for 10 min at 4 °C, followed by the removal of the supernatant. After the final wash, the RNA pellet was air-dried at room temperature and resuspended in 50 µL of DEPC-treated ultrapure water. The RNA concentration was estimated using 1 µL of the sample with a NanoDrop One^c^ Microvolume UV-Vis Spectrophotometer (Thermo Fisher Scientific, Waltham, MA USA). The remaining RNA was stored at −80 °C for future use.

### 4.9. Total RNA Sequencing

To ensure RNA quality before sequencing, 2100 BioAnalyzer technology (Agilent, Santa Clara, CA, USA) was used to assess RNA integrity, and only samples with an RNA integrity number (RIN) above 7. RNA libraries were then prepared by adding Illumina-specific adapters for sequencing on a HiSeq platform in a paired-end 150 bp configuration (Illumina, San Diego, CA, USA). Initially, read quality was assessed using FastQC v0.11.9. Reads were aligned to the human genome Grch38.p13 using STAR aligner v2.7.8, with GTF reference v.103 from ENSEMBL. After alignment, quality control was performed using MultiQC v1.13 to compile quality reports of the aligned sequences. Normalization and differential expression analyses were conducted using DESeq2 v1.36.0, allowing the identification of differentially expressed genes from the gene expression matrix.

### 4.10. cDNA Synthesis and qPCR Arrays

cDNA synthesis was performed using the SuperScript™ III First-Strand Synthesis System (Invitrogen™, Waltham, MA, USA) according to the manufacturer’s instructions, with a final elution volume of 40 µL of DEPC-treated water. To ensure that there was no genomic DNA contamination in the final cDNA product, the RNA sample was subjected to all steps except the addition of the reverse transcriptase enzyme. All samples were subjected to a second quality control using real-time PCR (qPCR) with SYBR Green reagent (Invitrogen, Waltham, MA, USA) and 500 nM of each GAPDH gene segment: forward primer 5′-GAGTCCACTGGCGTCTTCAC-3′ and reverse primer 5′-GCACTGTGGTCATGAGTCCTTC-3′. qPCR was performed on a QuantStudio 3 Real-Time PCR System (Applied Biosystems, Waltham, MA, USA) using the following thermal cycling conditions: (1) 50 °C for 1 min; (2) 95 °C for 2 min; and (3) 40 cycles of 95 °C for 15 s followed by 60 °C for 1 min. To evaluate transcriptional changes in ATC cells after treatment with hDPSC-EVs or the control for 72 h, a qPCR array was used, which included 92 genes involved in the molecular mechanisms of cancer (TaqMan™ Array Human Molecular Mechanisms of Cancer, Applied Biosystems™, Waltham, MA, USA). The array also included four candidates for endogenous control genes (*18s*, *GAPDH*, *HPRT1*, and *GUSB*). The plates were prepared according to the manufacturer’s instructions using 1 ng of cDNA (estimated based on the RNA concentration used for cDNA synthesis) and 10 μL of TaqMan^®^ Gene Expression Master Mix (Applied Biosystems, Waltham, MA, USA) per well. The plate was analyzed using a QuantStudio 3 Real-Time PCR System. Cycle threshold (Ct) values were obtained using Applied Biosystems 7500 Real-Time PCR System software (Version 2.3, Thermo Fisher, Waltham, MA, USA) and used to determine the log_2_ fold-change in each target gene, calculated in Excel (Office 2016, Microsoft, Redmond, WA, USA). Pathway enrichment was performed using all valid log_2_ fold-change values from the WebGestalt site (https://www.webgestalt.org, accessed on 4 June 2024), applying gene set enrichment analysis (GSEA) and the ‘pathway’ functional database.

### 4.11. Live/Dead™ Viability Assay

To evaluate the cytotoxic effects of hDPSC-EVs on ATC cell lines, the cells were cultured in 6-well plates (Corning^®^, Corning, NY, USA) at a seeding density of 6 × 10^4^ cells/well for 72 h treatments and 20 × 10^4^ cells/well for 120 h treatments. The cells were treated with 50 μg/mL of hDPSC-EV protein mass-relative or control. After the designated time, the cells were collected using the TrypLE™ Select Enzyme (Gibco™, Grand Island, NY, USA) and stained using the LIVE/DEAD™ Fixable Aqua Dead Cell Stain Kit (Invitrogen™, Waltham, MA, USA) according to the manufacturer’s instructions. Flow cytometry was performed using BD FACSCanto™ II (BD Bioscience, Franklin Lakes, NJ, USA) at 367/526 nm. Fluorescence calibration involved dead cell control (ATC cells subjected to heat shock) and an unstained control. For each sample, 1 × 10^4^ events within a gate were tracked using FSC/SSC. FlowJo™ 10 software (BD, Ashland, OR, USA) was used for data analysis, employing dead cell controls to establish live/dead cell gates (Figure S1B) and calculate the percentages of viable cells.

### 4.12. Cell Proliferation Assessment Using the CellTrace™ Violet Assay

To evaluate the proliferative profile of ATC cells after treatment with hDPSC-EVs, the CellTrace™ Violet assay (Invitrogen™, Waltham, MA, USA) was performed. This assay permanently labels cells with fluorescent dyes to track generation or division in vitro. In the CellTrace™ Violet assay, cells were stained with CellTrace™ Violet reagent (1 μL of dye per 1 × 10^6^ cells), according to the manufacturer’s instructions. A small number of cells were set aside to confirm staining efficiency. After confirmation, 1 × 10^5^ cells were fixed with 4% PFA for 15 min for the T0 (time zero) sample and an aliquot was set aside before staining as the unstained control. The remaining fresh, stained cells were seeded in triplicate for time points at 48 h, 72 h, 96 h, and 120 h for both treatments (control and 50 μg/mL of hDPSC-EV protein mass-relative). After cell adherence, the culture medium was replaced with a serum-free medium to synchronize the cell cycle before treatment. At each time point, the cells were harvested, fixed with 4% PFA for 15 min, and stored in PBS at 4 °C. At the end of the experiment, the cells were analyzed using a BD FACSCanto™ II flow cytometer at 405/450 nm. A total of 1 × 10^4^ events was acquired within a predefined gate using FSC/SSC for each sample. Data analysis was performed using FlowJo™ 10 software with the gate strategy demonstrated in Figure S1C, providing each replicate’s MFI. Statistical analyses were performed using GraphPad Prism 10.0.0 software.

### 4.13. Cell Cycle Assessment

To evaluate the impact of hDPSC-EV treatment on cell cycle phases, ATC cells were cultured in 6-well plates and subjected to a 24 h FBS deprivation period before treatment with hDPSC-EVs, as described in the ‘Live/Dead Viability Analysis’ section. Cells were fixed in 70% cold ethanol for 1 h, washed with PBS, and stained with 0.5 mL of FxCycle™ PI/RNase Staining Solution (Invitrogen, Waltham, MA, USA) for 30 min. Subsequently, the stained cells were analyzed using a BD FACSCanto II flow cytometer in triplicate, recording 1 × 10^4^ events per sample at 488/585 nm. Data analysis was conducted using the cell cycle analysis tool FlowJo™ 10 software with the gate strategy demonstrated in Figure S1D to calculate the percentage of cells in the G0/G1, S, and M/G2 phases. These percentages were subjected to statistical analysis using GraphPad Prism 10.0.0 software.

### 4.14. Cell Metabolism Assessment

To investigate the impacts of 72 h hDPSC-EV treatment on the metabolic and oxidative environment of ATC cells, we assessed changes in mitochondrial membrane potential (ΔΨm) using MitoTracker^®^ Green FM (Invitrogen™, Waltham, MA, USA) and evaluated reactive oxygen species (ROS) production using the ‘General Oxidative Stress Indicator’ CM-H2DCFDA (Invitrogen™, Waltham, MA, USA). The cells for these assays were seeded and treated as described for the viability assay. After 72 h of treatment, the culture medium was discarded and the MitoTracker^®^ Green FM stock solution was diluted with PBS to a final concentration of 75 nM. Cells were incubated under culture conditions for 20 min, washed with PBS, and collected. An unstained control without MitoTracker^®^ Green was also prepared for compensation using flow cytometry. All samples were promptly analyzed on a BD FACSCanto™ II in triplicate, with 1 × 10^4^ events analyzed per sample at 490/516 nm. The CM-H2DCFDA protocol was performed as described for MitoTracker^®^ Green, with the only differences being the reagent concentration (working concentration of 5 μM) and the dye incubation time (30 min). MFI values were used in GraphPad Prism 10.0.0 software for statistical analysis.

### 4.15. Migration Assessment

To evaluate the effect of hDPSC-EV treatment on the migration behavior of ATC cells, 1 × 10^4^ cells were initially seeded in 24-well plates and treated with 50 µg/mL of hDPSC-EV protein mass-relative or culture medium alone for 72 h. After this period, the culture medium was replaced with fresh medium containing only 1% fetal bovine serum (FBS) to restrict cell proliferation and facilitate the observation of migration. To initiate the migration assay, a scratch was made in each well using a P200 pipette tip, creating a clear ‘wound’ in the cell monolayer. Immediately after scratching, the plates were transferred to a TCS SP8 confocal microscope (Leica Microsystems, Wetzlar, Germany), and images were captured every 2 h for a 48 h period. The obtained images were analyzed using FIJI/ImageJ software, focusing on quantifying the wound closure area over time. This analysis allowed the evaluation of the potential of EVs to promote cell migration compared to the control. GraphPad Prism 10.0.0 software was used for statistical analysis.

### 4.16. Invasion Assay

This assay evaluates the ability of cells to migrate through a Matrigel^®^ layer using Transwell inserts with polycarbonate membranes and 8.0 μm pores (Corning^®^, Corning, NY, USA), following the methodology proposed by Pijuan et al. [37]. The procedure began by coating the upper chamber with 100 μL of Matrigel^®^ Matrix (final concentration of 300 μg/mL, Corning^®^, Corning, NY, USA) and allowing it to solidify overnight at 37 °C. The next day, 4 × 10^4^ cells pre-treated with 50 μg/mL of hDPSC-EV protein mass-relative for 72 h were seeded on top of the Matrigel in 300 μL of serum-free culture medium. Simultaneously, the lower chamber of the Transwell was filled with 800 μL of RPMI 1640 medium enriched with 10% FBS, which acted as a chemoattractant. After 24 h of incubation, non-invading cells were removed from the upper surface of the insert using a cotton swab, and the insert was washed with PBS. Cells that had migrated to the lower surface of the insert were fixed with 4% paraformaldehyde for 15 min and stained with 1 μg/mL of Hoechst 33,342 (Invitrogen™, Waltham, MA, USA), and visualized under a fluorescence microscope (Nikon Eclipse Ni with Nikon DS-Ri1 camera). Images of three fields per insert were captured using a 10× objective. The number of effectively invaded cells was quantified using ImageJ/FIJI software, as described by Pijuan et al. [37], and GraphPad Prism 10.0.0 software was used for statistical analysis.

## Data Availability

Further inquires can be directed to the corresponding authors.

## Supplementary Materials

The supporting information can be downloaded at: https://www.mdpi.com/article/10.3390/ijms26136443/s1.

## Abbreviations

The following abbreviations are used in this manuscript:

ATC	Anaplastic Thyroid Carcinoma
DAPI	4′,6-Diamidino-2-phenylindole
DEG(s)	Differentially Expressed Gene(s)
EV(s)	Extracellular Vesicle(s)
FBS	Fetal Bovine Serum
FDR	False Discovery Rate
GSEA	Gene Set Enrichment Analysis
hDPSCs	Human Dental Pulp Stem Cells
hDPSC-EV(s)	Extracellular Vesicle(s) Derived from Human Dental Pulp Stem Cells
MAPK	Mitogen-Activated Protein Kinase
MFI	Mean Fluorescence Intensity
MSC-EVs	Mesenchymal Stem Cell-Derived Extracellular Vesicles
NTA	Nanoparticle Tracking Analysis
PBS	Phosphate-Buffered Saline
PI	Propidium Iodide
qPCR	Quantitative Polymerase Chain Reaction
RNA-seq	RNA Sequencing
ROS	Reactive Oxygen Species
SD	Standard Deviation
ΔΨm	Mitochondrial Membrane Potential
