# Supplementary material for: Preclinical In Vitro Evaluation of Extracellular Vesicles from Human Dental Pulp Stem Cells for the Safe and Selective Modulation of Anaplastic Thyroid Carcinoma
Source: Int J Mol Sci. 2025 Jul 4;26(13):6443. doi: 10.3390/ijms26136443 (PMC12250338; doi:10.3390/ijms26136443)
Supplement: Supplementary file 1 [file ijms-26-06443-s001.zip › ijms-3681333-supplementary.pdf]

International Journal of
Molecular Sciences

MDPI

Supplementary Data

Preclinical In Vitro Evaluation of Extracellular Vesicles from Human Dental Pulp Stem Cells for the Safe and Selective Modulation of Anaplastic Thyroid Carcinoma

Anderson Lucas Alievi [1,2], Michelli Ramires Teixeira [1,2], Vitor Rodrigues da Costa [2,3], Irina Kerkis [2,3,*] and Rodrigo Pinheiro Araldi [1,3,4,*]

1 Postgraduate Program in Endocrinology and Metabology, Paulista School of Medicine of the Federal University of São Paulo (EPM/UNIFESP), São Paulo 04023-062, SP, Brazil; anderson.alievi@butantan.gov.br (A.L.A.); michelli.teixeira@butantan.gov.br (M.R.T.)
2 Genetics Laboratory, Butantan Institute, São Paulo 05503-900, SP, Brazil; vitor.rodrigues@butantan.gov.br
3 Department of Morphology and Genetics, Paulista School of Medicine of the Federal University of São Paulo (EPM/UNIFESP), São Paulo 04023-062, SP, Brazil
4 BioDecision Analytics Ltd., São Paulo 05713-510, SP, Brazil
* Correspondence: irina.kerkis@butantan.gov.br (I.K.); rodrigo.araldi@unifesp.br (R.P.A.)

Table S1. Complete information of antibodies used to characterize hDPSCs using flow cytometry.

Type	Target	Fluorophore	Diluition	Isotype	Manufacturer	Clone	Catalog no.	RRID
+	CD73	BB515	1:50	Mouse IgG1, κ	BD Biosciences	AD2	565110	AB_2739072
+	CD90	APC	1:50	Mouse IgG1, κ	BD Biosciences	5E10	559869	AB_398677
+	CD105	APC	1:50	Mouse IgG1, κ	BD Biosciences	266	562408	AB_11154045
-	CD14 [1]	PerCP	1:25	Mouse IgG2a, κ	Invitrogen	TuK4	MHCD1431	AB_10374157
-	CD34	APC	1:25	Mouse IgG1, κ	BD Biosciences	581	555824	AB_398614
-	CD19	APC	1:25	Mouse IgG1, κ	BD Biosciences	HIB19	555415	AB_398597
-	CD45	BB515	1:25	Mouse IgG1, κ	BD Biosciences	HI30	564585	AB_2869588
-	HLA-DR	BB515	1:25	Mouse IgG2a, κ	BD Biosciences	G46-6	564516	AB_2732846
IC	n/a	BB515	1:25	Mouse IgG1, κ	BD Biosciences	X40	564416	AB_2721017
IC	n/a	BB515	1:25	Mouse IgG2a, κ	BD Biosciences	G155-178	564515	AB_2869586
IC	n/a	APC	1:25	Mouse IgG1, κ	BD Biosciences	MOPC-21	555751	n/a

[1] Isotype Control not available from the manufacturer. n/a: non-available.

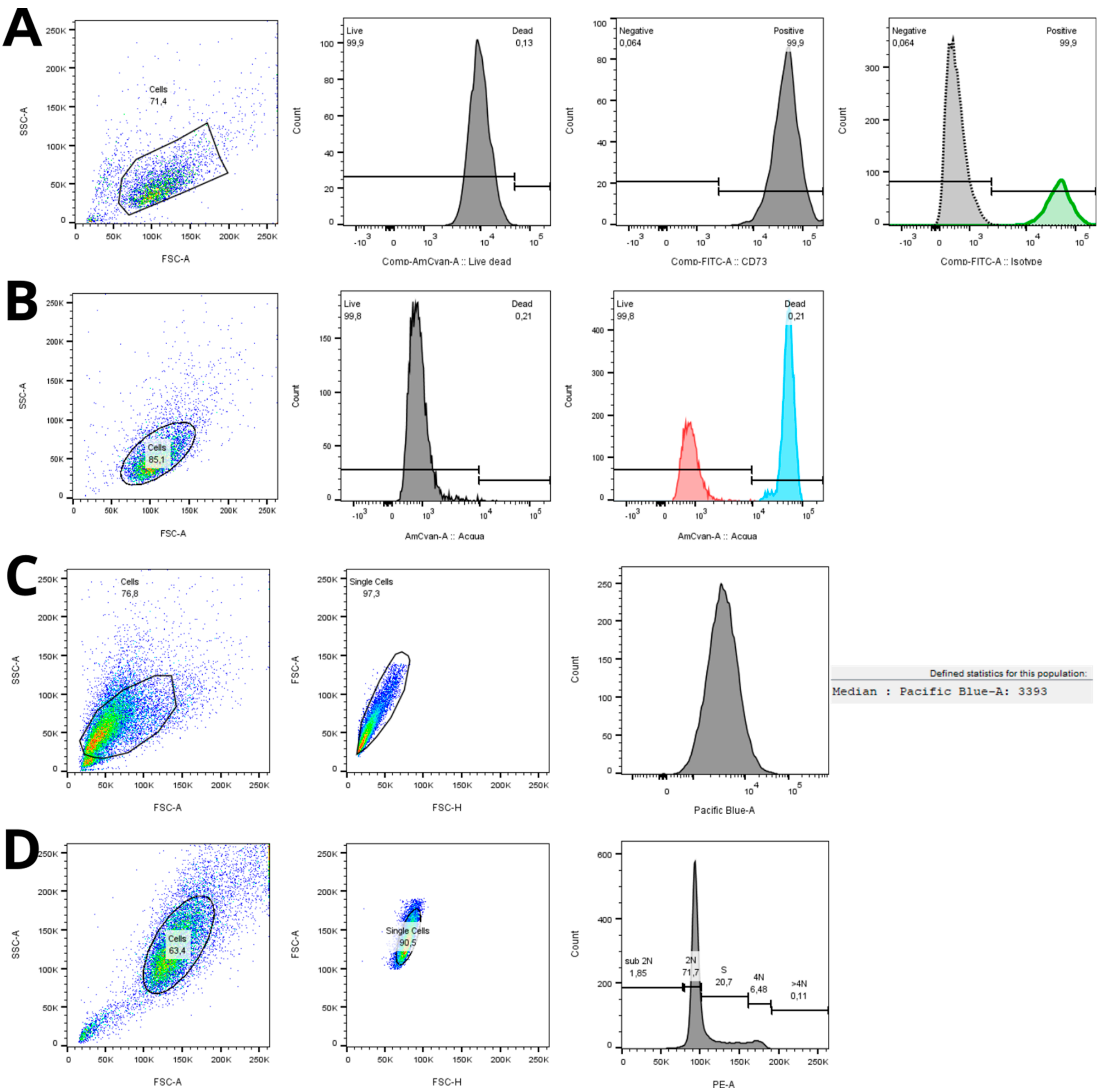

Figure S1. Gating strategy for flow cytometry analysis. (A) MSC Membrane Markers: The first plot (FSC-A vs. SSC-A) identifies and selects the cell population. Subsequently, the Live/Dead™ Viability Assay reagent was used to select only live cells. Each sample was then assessed for the percentage of marker expression compared to the corresponding isotype control. In the final plot, filled gray histograms represent isotype controls, while histograms with green or red lines represent antibody-labeled cells. (B) Live/dead cell analysis: An initial plot (FSC-A vs. SSC-A) was used to select the cell population. The second plot shows the distribution of live (low stained) and dead (stained) cells using predefined gates based on the death and viability controls. The final plot illustrates both the death control and the evaluated sample, indicating the percentage of live and dead cells in the population. (C) Cellular Proliferation: The first plot (FSC-A vs. SSC-A) was used to select the cell population. In the second plot (FSC-A vs. FSC-H), single cells were selected. The third plot shows the distribution of cells stained with CellTrace Violet proliferation dye. A decrease in fluorescence intensity over time indicates cell division. Fluorescence intensity was used to calculate proliferation rates. (D) Cell Cycle Assessment: The initial plot (FSC-A vs. SSC-A) was used to select the cell population. The second plot (FSC-A vs. FSC-H) selects single cells. The third plot displays

the cell cycle distribution based on DNA content: cells in the G0/G1 phase had the lowest DNA content (2N), S phase cells had intermediate DNA content, and G2/M phase cells had the highest DNA content (4N). The percentages of cells in each phase are shown.

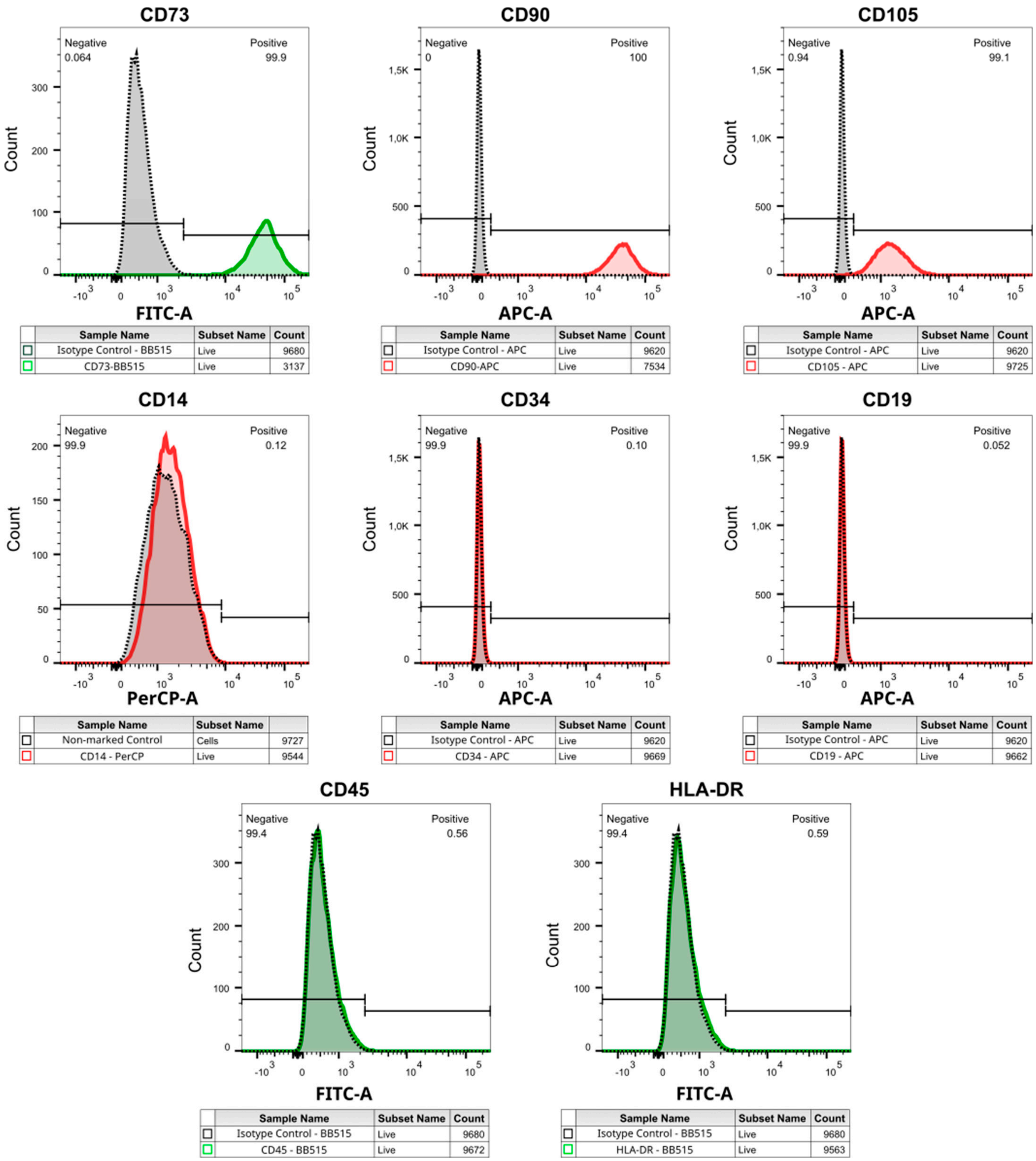

Figure S2. Immunophenotypic characterization of hDPSCs by flow cytometry. Histogram plots illustrate the expression of mesenchymal stem cell (MSC) surface markers CD73, CD90, and CD105 (positive markers) and the absence of hematopoietic cell markers (CD14, CD19, CD34, CD45) and major histocompatibility complex marker (HLA-DR) (negative markers) in hDPSCs. The filled grey histograms represent isotype controls or unmarked control cells.

A

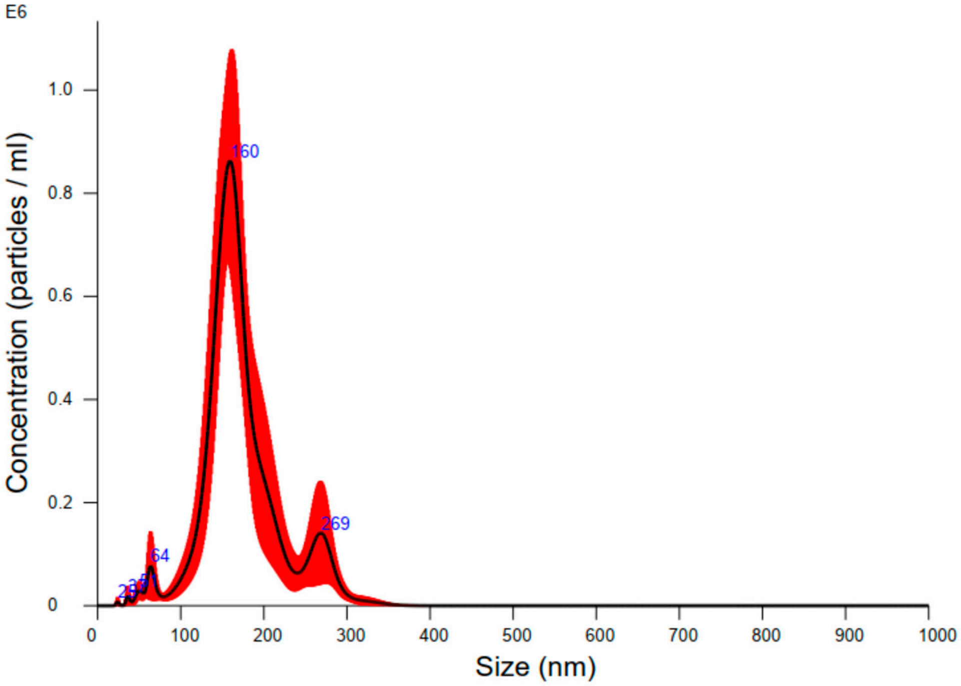

B

Stats: Mean +/- Standard Error	
Mean:	173.3 +/- 6.0 nm
Mode:	165.2 +/- 7.7 nm
SD:	43.5 +/- 5.5 nm
D10:	132.2 +/- 7.0 nm
D50:	166.3 +/- 6.4 nm
D90:	221.3 +/- 15.2 nm
Concentration:	5.64e+07 +/- 2.79e+06 particles/ml
**Dilution factor 1:20 = 1.13e+09 particles/mL**	3.1 +/- 0.2 particles/frame
	5.4 +/- 0.3 centres/frame

Figure S3. Size distribution and concentration analysis of hDPSC-derived EVs using nanoparticle tracking analysis (NTA). (A) The graph displays the particle size distribution profile of hDPSC-EVs, where the x-axis represents the size (nm) and the y-axis represents the concentration of particles/ml. The red curve indicates the particle-size distribution. (B) A table summarizing the statistical data obtained from the NTA analysis, including the mean, mode, standard deviation (SD), and percentiles (D10, D50, and D90) of the particle size distribution, as well as the concentration of particles in the sample. The dilution factor used for the analysis is also provided.

Table S2. Quality control of RNAseq reads using the FastQC/MultiQC software.

Sample	%GC	Phred score	*Read lenght* (pb)	*M Seq*
8505c_1_R1	48	36	150	25.8
8505c_1_R2	48	36	150	25.8
8505c_2_R1	48	36	150	22.5
8505c_2_R2	48	36	150	22.5
8505c_3_R1	53	36	150	69.6
8505c_3_R2	53	36	150	69.6
8505c_1_R1 + EVs	51	36	150	69.8
8505c_1_R2 + EVs	51	36	150	69.8
8505c_2_R1 + EVs	50	36	150	68.1
8505c_2_R1 + EVs	51	36	150	68.1
KTC2_1_R1	52	36	150	61.4
KTC2_1_R2	52	36	150	61.4
KTC2_2_R1	52	36	150	67.2
KTC2_2_R2	52	36	150	67.2
KTC2_1_R1 + EVs	52	36	150	64.0
KTC2_1_R2 + EVs	52	36	150	64.0
KTC2_2_R1 + EVs	48	36	150	57.5
KTC2_2_R2 + EVs	48	36	150	57.5

%GC: percentual of guanine and cytosine.

Table S3. qPCR gene expression using Molecular Mechanisms of Cancer array.

Genes	8505C	HTH83	KTC-2
*18S**	*		
*GAPDH**	*	*	*
*HPRT1**	*	*	*
*GUSB**			*
ABL1	0,199	0,7032	1,1886
AKT1	0,6369	-0,1503	0,1679
AKT2	0,4979	0,491	1,0024
APC	N/A	N/A	N/A
BAX	0,7973	0,2896	0,3183
BCAR2	0,1604	0,9753	0,5254
BCL2	N/A	N/A	N/A
BCL2L1	0,8235	0,1202	6,5415
BCL2L11	0,1671	-0,1632	0,0195
BID	0,7647	N/A	N/A
BRAF	N/A	N/A	N/A
CASP8	N/A	0,9334	0,2555
CASP9	0,9599	-0,1055	-0,0082
CCND1	-0,7164	0,3217	0,7523
CCND2	N/A	N/A	N/A
CCND3	0,6406	-0,0137	-0,0633
CCNE1	0,3772	-0,0023	-0,0624
CDC42	N/A	N/A	-0,4251
CDH1	N/A	N/A	N/A
CDK2	-0,3209	0,1438	-0,0815
CDK4	0,438	-0,0117	0,1821
CDKN1A	0,5087	0,001	0,2253
CDKN1B	-0,5847	-0,3043	0,1755
CDKN2A	-0,403	N/A	N/A
CDKN2B	N/A	N/A	N/A
COL1A1	N/A	N/A	N/A
CRK	-0,274	-0,7161	-0,3549
CTNNB1	-1,956	0,4916	-0,1141
CYCS	-0,3392	0,0807	-0,2448
DVL1	-0,0378	0,1095	-0,1947
E2F1	0,7288	-0,141	0,0539
EGFR	N/A	-0,1334	0,6211
ELK1	0,5036	0,1072	0,4085
ERBB2	0,5019	0,2214	0,3798
FADD	N/A	N/A	N/A
FAS	0,0501	N/A	0,2503
FASLG	N/A	N/A	N/A
FGF2	-0,8983	N/A	-0,5867
FN1	-2,5407	4,6814	4,0694
FOS	0,3013	0,0327	-0,1656
FYN	0,2775	0,0852	0,2902
FZD1	N/A	N/A	0,3182
GRB2	0,5532	-0,0209	0,1615
GSK3B	-0,2614	-0,0942	-0,0196
HGF	N/A	N/A	N/A

HRAS	0,5056	0,0189	-0,0971
IGF1	N/A	N/A	N/A
IGF1R	-0,3692	0,1199	0,637
ITGA2B	N/A	N/A	N/A
ITGAV	-0,8333	0,5771	1,2554
ITGB1	-0,7496	-0,1493	0,8417
ITGB3	0,0165	2,5968	1,2443
JUN	-0,5712	-0,057	-0,213
KDR	N/A	N/A	N/A
KIT	N/A	N/A	N/A
KRAS	N/A	N/A	0,0894
LEF1	N/A	-0,2993	0,5162
MAP2K1	0,8838	0,0495	0,2009
MAP3K5	N/A	N/A	1,0649
MAPK1	-0,2951	-0,0567	0,1529
MAPK14	-0,0109	0,0776	0,2417
MAPK3	0,2766	-0,0925	0,0217
MAPK8	N/A	-0,1547	0,1152
MAX	N/A	N/A	N/A
MDM2	N/A	N/A	-0,0647
MYC	0,5403	0,1701	-0,1269
NFKB1	N/A	N/A	0,2582
NFKB2	0,7574	0,0238	0,4304
NFKBIA	0,8103	-0,6208	-0,556
NRAS	-0,1919	-0,0956	-0,0032
PIK3CA	N/A	N/A	-0,6578
PIK3R1	N/A	N/A	0,9914
PTEN	N/A	N/A	N/A
PTK2	-0,1807	-0,1671	0,1503
PTK2B	1,3315	0,0368	-0,0578
RAC1	0,1126	-0,0998	0,1846
RAF1	0,1021	-0,151	0,1486
RB1	-0,4229	-0,2699	-0,3419
RELA	0,6665	-0,0052	0,2525
RHOA	0,6961	-0,1385	0,0276
SHC1	0,6128	-0,0565	0,1282
SMAD4	-0,0048	-0,1582	0,1993
SOS1	N/A	N/A	-0,0079
SPP1	N/A	-0,1652	-0,2023
SRC	0,5393	0,084	0,7012
TCF3	1,0193	-0,3574	0,255
TGFB1	0,8999	-0,0977	0,1516
TGFBR1	N/A	N/A	1,254
TGFBR2	-0,0791	-0,2646	0,2603
TP53	-4,2645	N/A	N/A
VEGFA	0,2407	-0,0409	0,2373
WNT1	N/A	N/A	N/A

Values are shown as log_2 fold-change (Log_2FC) in gene expression after 72 hours of hDPSC-EV treatment compared to untreated cells. *Housekeeping genes. N/A indicates genes not expressed or with undetectable levels in the specific cell line.

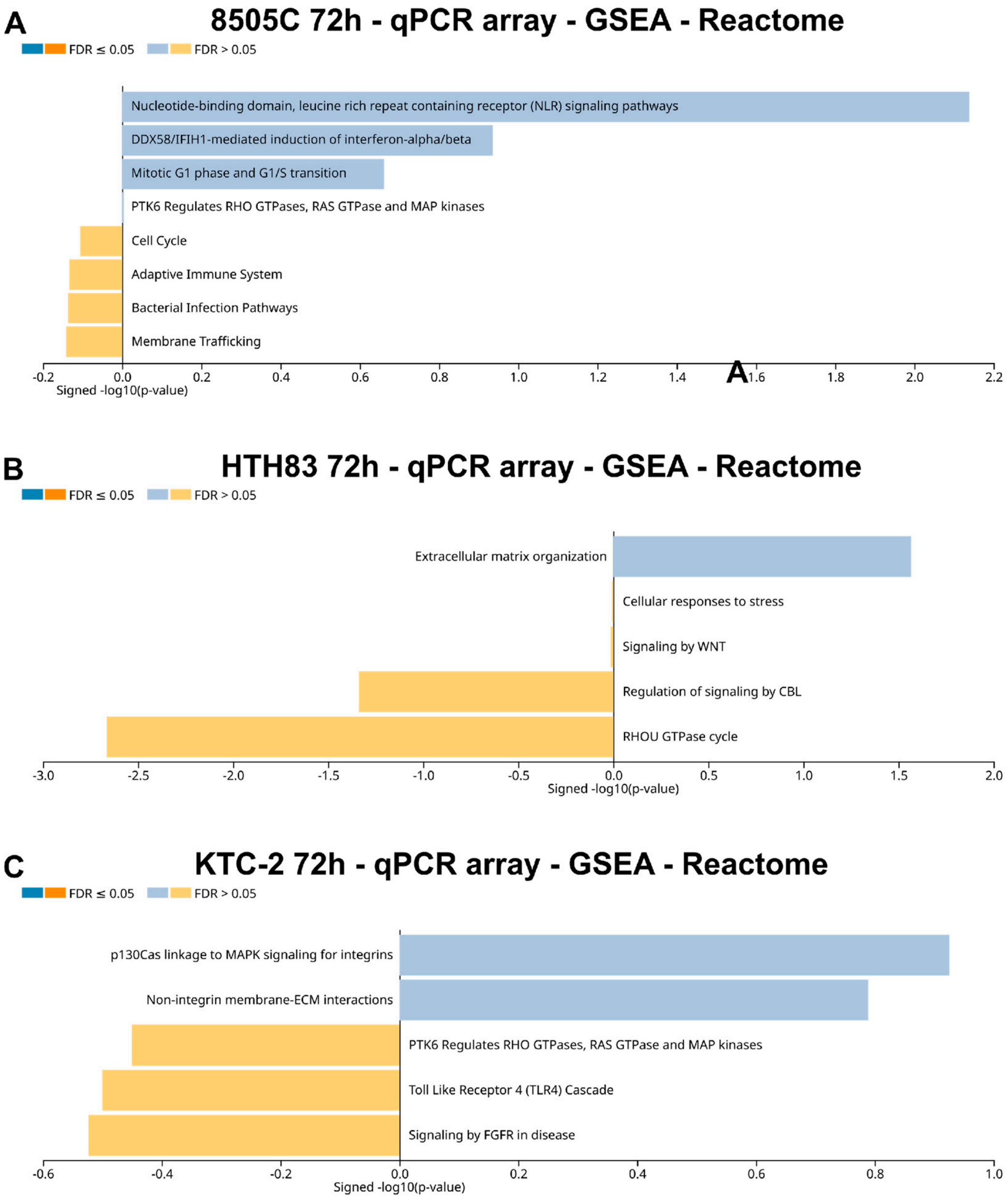

Figure S4. Pathway analysis using GSEA on qPCR data

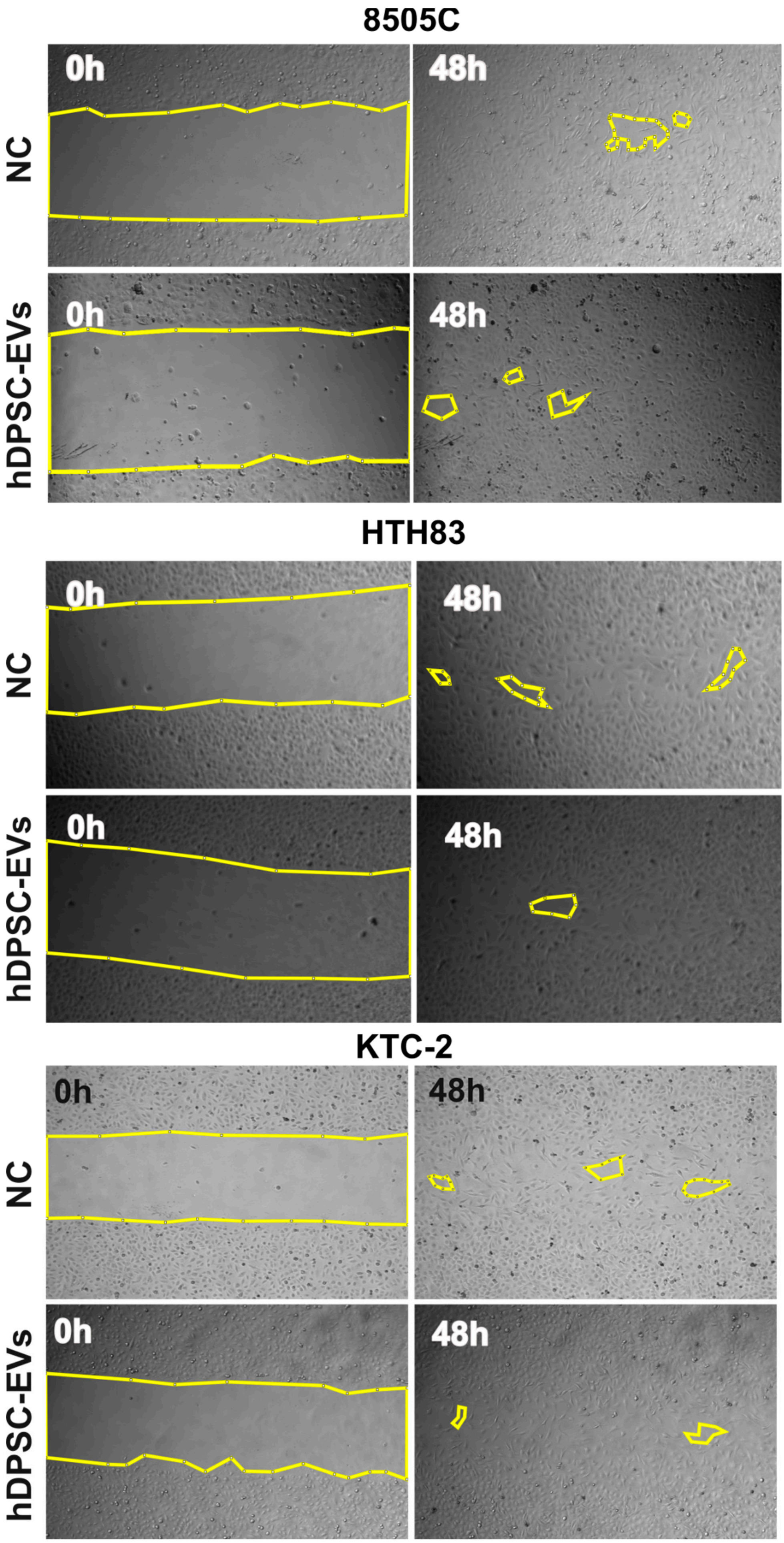
8505C
NC
hDPSC-EVs
0h
48h
HTH83
NC
hDPSC-EVs
0h
48h
KTC-2
NC
hDPSC-EVs
0h
48h

Figure S5. Representative images of Wound Healing assay

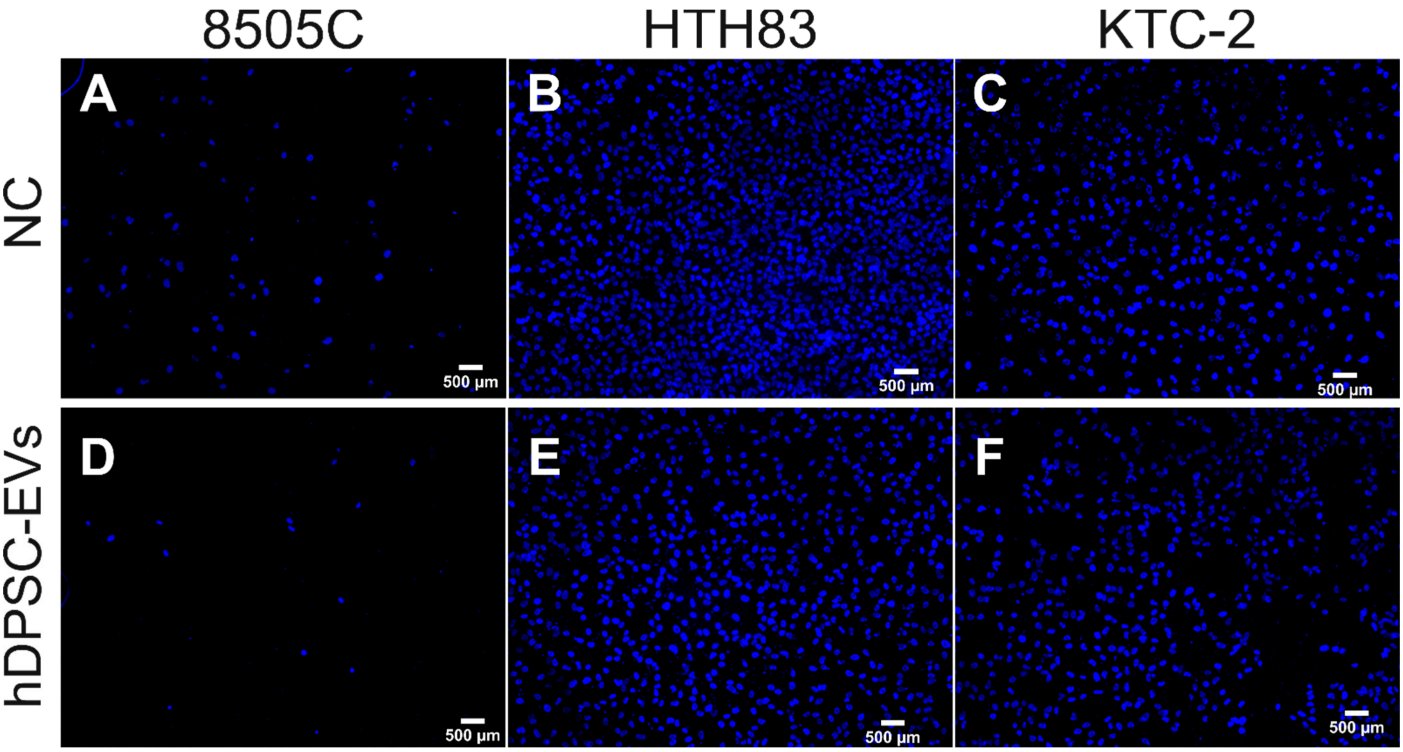

Figure S6. Representative images of Invasion assay
